# An Augmented Reality Technology to Provide Demonstrative Inhaler Technique Education for Patients With Asthma: Interview Study Among Patients, Health Professionals, and Key Community Stakeholders

**DOI:** 10.2196/34958

**Published:** 2023-03-02

**Authors:** Antonia O'Connor, Kelsey Sharrad, Charmaine King, Kristin Carson-Chahhoud

**Affiliations:** 1 Adelaide Medical School The University of Adelaide Adelaide Australia; 2 Department of Respiratory and Sleep Medicine Women's and Children's Hospital Adelaide Australia; 3 Translational Medicine and Technology Group Australian Centre for Precision Health University of South Australia Adelaide Australia; 4 Cancer Research Institute University of South Australia Adelaide Australia; 5 South Australian Health and Medical Research Institute Adelaide Australia

**Keywords:** augmented reality, asthma, disease management, smartphone, inhaler technique, mobile phone

## Abstract

**Background:**

Many people with asthma use incorrect inhaler technique, resulting in suboptimal disease management and increased health service use. Novel ways of delivering appropriate instructions are needed.

**Objective:**

This study explored stakeholder perspectives on the potential use of augmented reality (AR) technology to improve asthma inhaler technique education.

**Methods:**

On the basis of existing evidence and resources, an information poster displaying the images of 22 asthma inhaler devices was developed. Using AR technology via a free smartphone app, the poster launched video demonstrations of correct inhaler technique for each device. In total, 21 semistructured, one‐on‐one interviews with health professionals, people with asthma, and key community stakeholders were conducted, and data were analyzed thematically using the Triandis model of interpersonal behavior.

**Results:**

A total of 21 participants were recruited into the study, and data saturation was achieved. People with asthma were confident with inhaler technique (mean score 9.17, SD 1.33, out of 10). However, health professionals and key community stakeholders identified that this perception was misguided (mean 7.25, SD 1.39, and mean 4.5, SD 0.71, for health professionals and key community stakeholders, respectively) and facilitates persistent incorrect inhaler use and suboptimal disease management. Delivering inhaler technique education using AR was favored by all participants (21/21, 100%), particularly around ease of use, with the ability to visually display inhaler techniques for each device. There was a strongly held belief that the technology has the capacity for improving inhaler technique across all participant groups (mean 9.25, SD 0.89, for participants; mean 9.83, SD 0.41, for health professionals; and mean 9.5, SD 0.71, for key community stakeholders). However, all participants (21/21, 100%) identified some barriers, particularly regarding access and appropriateness of AR for older people.

**Conclusions:**

AR technology may be a novel means to address poor inhaler technique among certain cohorts of patients with asthma and serve as a prompt for health professionals to initiate review of inhaler devices. A randomized controlled trial design is needed to evaluate the efficacy of this technology for use in the clinical care setting.

## Introduction

### Background

Asthma is a chronic respiratory disease affecting 1 in 9 Australians and 262 million people globally [1,2]. Current asthma management guidelines recommend that all patients are provided with guided self-management education [3]. This includes monitoring of symptoms and lung function; having a written asthma action plan; and regular review by a health professional, which may involve the use of pamphlets with pictures and demonstrations of inhaler devices [3,4]. However, the literature suggests that patients who access health professional support do not necessarily receive an explanation about their disease or correction of management missteps during these encounters [5-7]. A study by AL-Jahdali et al [8], found that >50% of patients with asthma had no formal education about the disease, and a further 40% had obtained no official education about the medications or inhaler devices from any health professionals. This is evidenced by an estimated 70% to 80% of people with asthma being unable to use their inhaler medication correctly [9]. Common mistakes in inhaler technique include inability to hold the breath long enough, breathing in very deep or not breathing sufficiently deep, and inability to coordinate inhaler use [10-13]. Preventable consequences of poor understanding of asthma and inhaler technique include treatment failure, leading to poor clinical outcomes, increased health care use, increased morbidity, high medication dosages, and poor quality of life [14-17].

Improvement in poor disease control can occur through review of inhaler technique, with health professionals correcting mistakes as they observe [10-12]. However, they may not be able to provide this level of support [6,7,18,19]. Health professionals are time poor, are overburdened, and have variable uptake of evidence-based guidelines in clinical practice [5-7,20]. Literature also suggests that health care professionals may not have adequate knowledge of asthma and inhaler technique themselves [17]. A systematic review by Plaza et al [21] analyzed 55 studies with >6300 participants identified as health care professionals, and results showed that only 15.5% of participants were considered to be proficient in inhaler technique. Furthermore, a study by Basheti et al [6] explored 200 health professionals and determined that there was significant association between poor asthma knowledge and inhaler technique. In addition, perceptions held by patients with asthma about health professionals can affect the range of mistakes being made and the amount of information available to them [22,23]. Qualitative studies indicate that some patients perceive general practitioners (GPs) as the only avenue of support or believe that alternate health professionals, particularly pharmacists, are unable to deliver medication advice [22]. A study by Cheong et al [22] interviewed 47 patients with asthma to determine how their perceptions and choices may affect multidisciplinary care. In particular, they looked at perceptions that participants held on the role of health professionals, convenience of accessing health advice, and asthma itself [22]. Furthermore, many people with asthma who perceive themselves to be adept in using their inhaler, make more or as many mistakes as those identifying as less confident [23,24]. Contributing to confusion, in Australia, people with asthma may use ≥1 of the 22 different inhaler devices available, depending on the device they are prescribed; therefore, they would need to master 1 of 6 different techniques [25]. A way to address poor inhaler knowledge and technique and increase use of prescribed medications is through improving education for patients and health care professionals [15,17].

A simple stepwise video demonstration may enhance education, as proper inhaler technique may not be portrayed sufficiently from manufacturer leaflets [26,27]. Advantages of multimedia education are noted from as early as 1983 and include the following: takeaway resources, allowing independent application; entertaining audio-visual for patients; and a modality that is less reliant on those with limited literacy skills [27-30]. A review by Abed et al [27] discovered 3 asthma studies that found multimedia to be useful for asthma education, with participants in 10 of the 20 included studies showing improvement following video-assisted patient education. Studies assessing the effect that multimedia and technology have on inhaler technique showed improvement in skill and knowledge following short-term and long-term interventions [27,31-35]. Studies have also shown multimedia to be effective for improving self-efficacy in children with asthma and their caregivers [36,37].

Technology known as augmented reality (AR), used as interactive print (an innovative medium allowing dissemination of educational advice), allows for digital enhancement of paper-based resources. AR technology can overcome issues of limited health literacy [38,39]; allow for tailoring to individual populations (eg, age and language); increase engagement [40]; increase accessibility of education [41]; and allow for real-time updates of content, as new evidence becomes available.

### Objective

Although multimedia has shown to be able to improve inhaler technique compared with traditional standard print, use in the form of AR has not been tested before. In addition, as with any new technology or innovation, it is necessary to ensure that it meets the demands of the main user—for example, health professionals, patients with asthma, and key community stakeholders such as the Asthma Foundation of South Australia. Therefore, the aim of this study was to obtain the perspectives of health professionals, patients with asthma, and key community stakeholders on the feasibility of innovative technology in the form of AR, particularly, current inhaler technique education level, technology use level, and potential of AR.

## Methods

### Ethics Approval

The Human Research Ethics Committee of The Queen Elizabeth Hospital (TQEH) granted both ethics approval (July 13, 2016) and governance approval (July 29, 2016; Q20160614). Acceptance of approval (July 25, 2016) and relevant insurance (July 26, 2016) from the Human Research Ethics Committee and Legal and Risk Branch of The University of Adelaide were also obtained.

### Development of Paper-Based Resource

Following review of research evidence, in addition to recommendations from national and international asthma guidelines [8,9,12,24,25,42], a prototype poster (Multimedia Appendix 1) for patient information around inhaler technique was developed. A senior scientist on the Medical and Scientific Advisory Committee for the Asthma Foundation of South Australia examined the prototype for relevance and applicability for use by health professionals and patients with asthma. A digital version of the poster was uploaded to a web-based Layar server and superimposed with educational inhaler technique videos provided by the Lung Foundation of Australia [42]. After publishing the AR version of the poster, the free Layar application could be used to scan printed versions of the paper-based poster, thus triggering pattern recognition. Then, the educational videos were activated, thereby initiating the print-based poster to *activate* on a smartphone screen. Layar allows digital media to be added to paper-based resources, augmenting them and providing the observer with a direct view of correct inhaler technique video demonstration [43]. The free smartphone app allowed demonstration of the technology throughout interviews and further aided in gaining perspectives from participants and potential users.

### Participant Enlistment in the Study

Inclusion criteria for all participants were based on the current gap in the literature for inhaler technique. Participant recruitment commenced in August 2016 and continued until sufficient data saturation was achieved in September 2016. All potential participants were contacted to assess willingness of participation. Participant information sheets and consent forms were distributed following agreement for consideration, with 24 hours allowed to discuss enrollment in the study with friends and family members. Interviews were scheduled following reading and understanding of participant information sheets and verbal and written agreement of participation.

### Recruitment of Health Professionals

Health professionals were recruited through existing contacts of the Respiratory Medicine Department at TQEH, SA Health, and Asthma Foundation of South Australia. Health professionals intended for interviewing included respiratory specialists, GPs, respiratory nurses, and pharmacists, who are currently consulting patients with asthma [5,6,44,45]. Overall, 8 semistructured, one-on-one interviews (n=2, 25% with each profession) were considered feasible to reach data saturation, inclusively with patient with asthma and key community stakeholder interviews [46]. Health professionals who did not meet the inclusion criteria were excluded from participation. Specific health professional inclusion criteria comprised the following:

1. Providing consultation or assistance for patients with asthma over a minimum 12-month period, with at least one consult per week or equivalent

### Recruitment of Patients With Asthma

Recruitment of patients with asthma occurred through existing contacts throughout the Respiratory Department at TQEH via respiratory specialists. Semistructured, one-on-one interviews with 50% (3/6) male patients and 50% (3/6) female patients were considered feasible for data saturation, inclusively with health professionals and key community stakeholders [46]. Patients with asthma were recruited through purposive selection ensuring diversity of demographic characteristics and, in particular, sourcing patients who experience difficulty with inhaler technique [47]. For example, recruiting someone who is regularly admitted to hospital for inadequate asthma management, at least once per year, compared with someone with well-controlled asthma [9]. Inclusion criteria for patients with asthma comprised the following:

1. Aged >18 years
2. Formal diagnosis of asthma
3. No diagnosis of chronic obstructive pulmonary disease (COPD)

Patients with COPD can use different inhalers to manage their condition compared with patients who purely have asthma [25]. Therefore, to avoid confusion, people with COPD-asthma overlap syndrome were excluded.

### Recruitment of Key Community Stakeholders

In total, 4 key community stakeholders, identified through existing contacts of the principal investigator, were considered for recruitment from the Asthma Foundation of South Australia. They were chosen because their key role in delivering resources and training is particularly focused on health professionals and patients with asthma. Semistructured, one-on-one interviews were conducted with participants involved in asthma education including asthma researchers, policy makers, and asthma health workers. Overall, 4 interviews were considered feasible in obtaining data saturation, inclusively with health professional and patient interviews. Key community stakeholder inclusion criteria comprised the following:

1. Involved with people with asthma or had community consultation capacity for a minimum 12-month period over the past 5 years

### Data Analysis

Interview data were deidentified for coding, analysis, and publication purposes. Participants were also given the opportunity to review their transcript before publication and dissemination.

NVivo Pro 11 (QSR International) allowed qualitative data (interview transcripts) to be analyzed by 2 independent coders. Analysis and coding occurred under thematic categories based on the Triandis model of interpersonal behavior (explained in the following sections), and they were further divided into positive and negative participant perspectives to reduce confirmation bias [48].

Scores on 10-point Likert scales assessing attitudes, knowledge, perceptions, and beliefs of inhaler use and education were collected during interviews and expressed as means and SDs. Other quantitative data including demographics, inhaler use, asthma education, and more were also collected. Use of both qualitative and quantitative data enhances triangulation of information, which is important to improve strength and dependability of findings [49]. Moreover, it allows comparison of the 3 participant groups’ responses through 3 levels of the Triandis model, leading to improved reliability and validity [49,50].

### Theoretical Underpinning

The Triandis model of interpersonal behavior belongs to a school of cognitive models and considers intentions and habits as immediate precursors of behavior. Both are influenced by facilitating conditions [50]. According to Triandis, facilitating conditions including social and affective factors and rationale considerations (eg, incorrect inhaler technique causing decreased asthma management, leading to patients stopping the use of their inhalers) influence intentions [51]. This can indicate that no action is either fully deliberate or automatic, and therefore, behavior is believed to be influenced by intentions, habits, and facilitating conditions. The impact of this can be further limited by both emotional and cognitive function [50].

**Figure 1:**
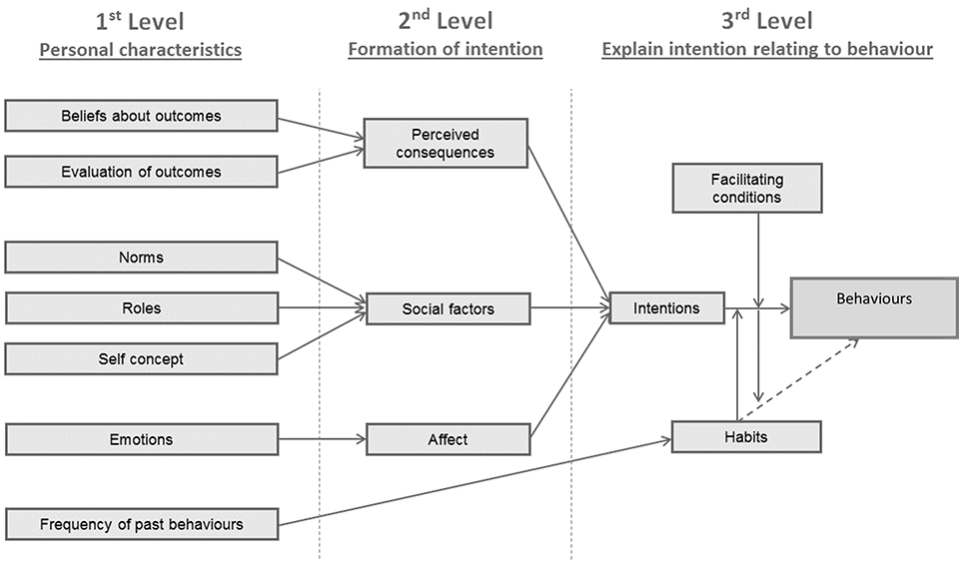
Modified representation of the Triandis model of interpersonal behavior by Egmond and Bruel [48,52].

An alternative way to consider the influences within the Triandis model is the trilevel explanation developed by Egmond and Bruel [48]. The study applied this form of the model throughout [48]. The model starts with investigating the behavior (inhaler technique) itself and working backward from that point. The third level explains intentions when influenced by facilitating conditions, that is, difficulty in understanding written instructions. It also explains habits such as using inhaler incorrectly to predict whether an individual will perform a particular behavior. The second level considers how cognition, affect (being pure emotion), and personal normative beliefs influence the creation of intentions regarding a specific or general behavior. Finally, the first level relates to personal characteristics that focus on past experiences, which shape perceived consequences, affect, and social factors related to behavior. Ultimately, the Triandis model offers a framework that provides explanations to understand complex human behaviors, particularly those influenced by affective and social factors (Figure 1).

## Results

### Overview

A total of 21 participants were recruited into the study, and data saturation was achieved. The sample consisted of 33% (7/21) male patients and 62% (13/21) female patients, aged between 21 and 64 years. The characteristics of all participants are reported in Table 1; however, raw age and gender information has been removed for confidentiality purposes.

**Table 1:** Participant characteristics.

Participant groups and IDs			Occupation		Education		Experience (years)		Number of asthma consultations		Education provided in consultation			Access to smartphone or device
**HPs^a^**														
	HP 1	Respiratory specialist		FAACP^b^—postgraduate degree		7		0-3 per day		Briefly			Yes	
	HP 2	Respiratory specialist		Bachelor of medicine and surgery		4		2 per month		Yes			Yes	
	HP 3	General practitioner		University degree		32		1-2 per day		Sometimes			Yes	
	HP 4	General practitioner		University degree		6		1-2 per day		Not always			Yes	
	HP 5	Respiratory nurse		Postgraduate degree		20		2-5 per month		Yes			Yes	
	HP 6	Respiratory nurse		University degree		15		15-20 per month		Yes			No	
	HP 7	Pharmacist		University degree		6		1 per day		Yes			Yes	
	HP 8	Pharmacist		University degree		10		2 per day		Yes			Yes	
**APs^c^**														
	AP 1	Laborer		Year 11		3		2-3 per year		Yes			Yes	
	AP 2	Research officer		TAFE^d^		19		1 per 1-2 years		No			Yes	
	AP 3	Senior medical scientist		Postgraduate diploma		8		1 per year		Yes			Yes	
	AP 4	Physician		Bachelor of medicine and surgery		29		1 per 1-2 years		No			Yes	
	AP 5	Baker		TAFE diploma		22		2 per month		Sometimes			Yes	
	AP 6	Medical scientist		Doctor of Philosophy		15		2 per year		Yes			Yes	
	AP 7	Home duties		Year 9		43		N/A		No			No	
	AP 8	Professional football player		Double degree in business management		23		4 per year		Yes			Yes	
**KCSs^e^**														
	KCS 1	Chief executive		Master’s degree		N/A^f^		N/A		N/A		Yes		
	KCS 2	Health services manager		N/A		N/A		N/A		N/A		Yes		
	KCS 3	Health promotion coordinator		Bachelor’s degree		9		2 per month		Yes		Yes		
	KCS 4	Health promotion coordinator		Bachelor’s degree		6		6-12 per day		Yes		No		

^a^HP: health professional.

^b^FRACP: Fellow of the Royal Australasian College of Physicians.

^c^AP: patient with asthma.

^d^TAFE: Technical and Further Education.

^e^KCS: key community stakeholder.

^f^N/A: not applicable.

### Positive Perspectives to Inhaler Technique Technological Interventions

#### First Level of Triandis—Personal Characteristics

A patient’s normal inhaler technique behavior is reviewed by health professionals. In particular, respiratory nurses and respiratory specialists pursued this evaluation before critique of inhaler technique. Hence, there appears to be a standard approach taken by health professionals when evaluating patient inhaler use; however, all health professionals (21/21, 100%) indicated this does not always occur. An approach used by health professionals involves observing current technique before evaluating practice to ensure that medication is administered correctly. Overall, 2 respiratory specialists (HP 1 and HP 2) and 2 GPs (HP 3 and HP 4) reported involving pharmacists, respiratory nurses, and key community stakeholders in providing correct inhaler technique education. This is conducted internally and externally from the clinic or hospital, thus allowing more time to consult patients with asthma in a more comfortable setting:

I will get them to show me what they do first and then I will demonstrate...the correct technique if it’s something they are already on. If it’s something they’ve never used I will demonstrate first and then I will get them to show me how they do it.

HP 5

In an inpatient setting they all will be educated by a pharmacist.

HP 2

#### Second Level of Triandis—Formation of Intention

Introduction of AR to help improve asthma inhaler technique education was perceived positively. All participants (21/21, 100%) perceived that it would have positive impact on improving inhaler technique. However, there was a divergence in views among groups, with all except 5 patients with asthma (5/8, 63%) perceiving that it may require further support resources such as written information on how to use the technology. Participants believed this would be particularly the case for older users. Furthermore, 25% (2/8) of the patients with asthma agreed that the technology would be a great resource for children aged ≥7 to use, owing to their perceived high level of interest in technology. Health professionals perceived that the technology would be particularly useful as an inhaler initiation tool for new users and a helpful refresher for those who may need to update their technique. All key community stakeholders and patients with asthma agreed that it would be preferred as a refresher tool. Attention was further drawn to ease of technology use, and all participants (21/21, 100%) agreed that it would be useful throughout a patient’s time with the disease. Furthermore, all participants (21/21, 100%) provided positive responses regarding practicality of the technology; however, they questioned if potential operators would use it. All 8 patients with asthma who were interviewed agreed that they would use it; however, they could only report hope that others would:

It’s a good start...cause that’s the question I’m asking myself...how correct am I in teaching the patient.

HP 6

On initial diagnosis I would imagine...or when they are sort of unsure about what to do then they could go back and refresh every so often to make sure their actually doing it correctly.

KCS 3

#### Third Level of Triandis—Explain Intention Relating to Behavior

Health professionals and key community stakeholders believe that habits and intentions play crucial roles in how a patient learns the behavior of inhaler technique. In particular, 25% (2/8) of the pharmacists agreed that most patients with asthma have the right intentions. However, throughout a technique checkup, some demonstrate incorrect inhaler technique that appears to have developed from previous habits. In contrast, all the patients with asthma who were interviewed (8/8, 100%) perceived themselves to be using their inhalers correctly, leading to development of good and bad habits in relation to inhaler use. All patients with asthma (8/8, 100%) agreed that they were questioning their confidence in using their inhaler correctly following the interview. Furthermore, introduction of AR technology was believed to be a facilitating condition. All participants (21/21, 100%) perceived that it would help to improve inhaler technique and reassure all potential handlers to use their inhalers correctly:

I think some customers are afraid...to ask me again if they are unsure about something. The first time they are very receptive and...appreciate that you spend the time...I think they’re a bit embarrassed about asking again.

HP 8

People don’t have to be able to read information and they’ve got a visual cue and they can come back and watch the demo again.

HP 5

There’s people that that take it seriously and want to get the best care, I think they will find that very useful.

AP 6

### Negative Perspectives to Inhaler Technique Technological Interventions

#### First Level of Triandis—Personal Characteristics

The study by AL-Jahdali et al [8] was mentioned during the interviews to determine how the results of the study made participants feel. Responses indicated that health professionals were aware of deficient education about asthma and inhaler devices. However, the health professionals also reported this absence being identified when concerns or problems with technique were raised by patients during consultation. In particular, 25% (2/8) of the health professionals, through their experience in practicing in a hospital, learned to question patients to understand what information was required. Furthermore, the 25% (2/8) of the health professionals believed that with time restraints and other health concerns taking priority, inhaler technique and asthma education were often forgotten or missed during consultation. In total, 25% (2/8) of the pharmacists added time as a barrier to demonstrating correct inhaler technique. Moreover, pharmacist’s evaluation of outcomes determined that there is a lack of notification from other health professionals and patients regarding when inhaler technique education is necessary. This leads to patients with asthma, in particular, 38% (3/8) of those interviewed, conceptualizing that they were never educated about the disease or inhaler devices until a serious event, hospitalization, or exacerbation had occurred:

I think we’ve all been guilty of just prescribing someone...a device...and just assuming that the patient’s going to read the instructions...when your pushed for time it’s a bit like, just make sure you read the instructions...and if you have any questions then contact me.

HP 3

I even still don’t fully understand the scientific side of asthma and the disease...I was basically just given a puffer and told this will fix it...

AP 2

#### Second Level of Triandis—Formation of Intention

A particular question regarding whose responsibility it is to deliver correct inhaler technique education was put forward throughout the interviews. Health professionals and key community stakeholders perceived it to be a combined effort from all involved individuals to consequently improve inhaler technique; however, this does not always occur. Interviewed patients with asthma had alternate perspectives. For example, they perceived GPs or respiratory specialists to be responsible for delivery of the education. This indicates that most patients do not perceive there are other available sources for education, which consequently leads to outcomes where patient problems are not being resolved, and intentions become negative:

I s’pose it’d be...respiratory doctors.

AP 1

I’ve only ever been to see my GP about my asthma...I expect it would be my GP.

AP 2

Well it’s combined responsibility...if they had some education at the GPs or the practice nurse that does education, then again at the pharmacists...they come into us and have more education hopefully we can get them on the right path.

KCS 3

#### Third Level of Triandis—Explain Intention Relating to Behavior

Barriers perceived by interviewed participants include bad habits that were formed many years ago and patients believing themselves to be proficient with their inhalers, but upon observation, using it incorrectly. Another barrier is with the technology itself. Although interviewed participants agreed that they would use it if they had access to it, many health professionals (8/8, 100%) believed that some patients would not be able to work with the technology. Therefore, they perceived that they would be the ones to implement the technology, further reducing consult time. In particular, a health professional mentioned concerns about the technology initially requiring internet until the video was downloaded, as not all patients with asthma have or can access this. Another health professional mentioned it being reliant on smartphones or other capable devices and not all patients with asthma having access to technology. A patient with asthma did not have a smartphone and agreed it was problematic:

One in cognitive function, memory, language fluency, literacy. So these are all potential barriers.

KCS 4

They understand but it’s their condition...

HP 6

I think one of the challenges for us in particular is that people generally don’t take the asthma seriously.

KCS 2

Not having smartphone maybe, this is dependent on a smartphone.

HP 1

I haven’t got the technology.

AP 7

Additional findings related to attitudes, knowledge, and beliefs around inhaler technique and technology in the form of AR, for each of the 3 levels of behavior, is reported in Multimedia Appendix 2.

### Questionnaire Data

Similarities were observed between quantitative and qualitative results among the 3 groups and 3 levels of the Triandis model on triangulating the results. There is a strongly held belief that the app or technology has the capacity for improving inhaler technique. Furthermore, it is believed that all users of the technology would benefit by using it, owing to the importance of patient inhaler technique.

As per the evidence in Table 2 and responses from the qualitative results, patients with asthma are very confident with their inhaler technique, which is represented by the mean score of 9.17 (SD 1.33) out of 10. However, health professionals and key community stakeholders believe that patients with asthma are typically not confident (mean 7.25, SD 1.39, and mean 4.5, SD 0.71, for health professionals and key community stakeholders, respectively).

Further adding to this incongruency, patients with asthma do not believe that they experience stress, anxiety, or worries when using their inhalers (mean 1.83, SD 2.51), whereas health professionals and key community stakeholders report that patients with asthma experience stress, are anxious, or report being worried when using their inhaler device (mean 5.38, SD 1.19, and mean 5, SD 1.41, respectively). Frequency of smartphone technology use is another instance where a divergence in views among groups can be observed, as shown in Table 2. Health professionals and key community stakeholders see themselves using the technology more often (mean 7.63, SD 0.92, and mean 8.25, SD 0.35, respectively) than the patients with asthma (mean 5.17, SD 1.75). The final discrepancy reported among the 3 groups was regarding the amount of education and help available for patient’s inhaler use. Patients with asthma believed that there was sufficient education or help available, with mean score of 6.5 (SD 2.71), whereas health professionals and key community stakeholders believed that there could be more education (mean 5.5, SD 2.07, and mean 5.5, SD 0.71, respectively). Additional findings related to attitudes, knowledge, and beliefs around inhaler technique for each of the 3 groups of participants are reported in Table 2*.*

**Table 2:** Quantitative questionnaire variables separated by the 3 levels of Triandis identifying participant attitudes, knowledge, and beliefs around inhaler technique^a^.

Levels and questionnaire variables			Health professionals, mean (SD)		Patients with asthma, mean (SD)		Key community stakeholders, mean (SD)
**1—Personal characteristics**							
	Sufficient education or help available regarding patient inhaler use	5.5 (2.07)		7.83 (2.71)		5.5 (0.71)	
	Technology will be helpful for inhaler technique	9 (0.93)		9.17 (1.33)		9.5 (0.71)	
	Health care professionals benefit from technology such as this	8.88 (0.64)		9.5 (1.22)		9.5 (0.71)	
**2—Formation of intention**							
	Patients experience stress, anxiety, or worries when using their inhaler	5.38 (1.19)		1.5 (2.51)		5 (1.41)	
	The technology has the capacity to improve inhaler technique	9.25 (0.89)		9.83 (0.41)		9.5 (0.71)	
	Importance of patient’s correct inhaler technique	9.88 (0.35)		9.67 (0.52)		10 (0)	
**3—Intention relating to behavior**							
	Patient’s confidence when using their inhaler	7.25 (1.39)		9.17 (1.33)		4.5 (0.71)	
	Repeated use of technology	7.63 (0.92)		4.67 (1.75)		8.25 (0.35)	

^a^Mean=average score (out of 10).

## Discussion

### Principal Findings

#### Overview

This study used a qualitative interviewing approach to investigate the potential use of AR to improve upon existing education for asthma inhaler technique. All participants (21/21, 100%) were from Adelaide, South Australia, with varying occupations, gender, age, experience of teaching or using an inhaler, and technology use. Results from all participants reflect the importance of correct inhaler technique education, with three key factors being identified: (1) health professionals’ and patients’ confidence with inhaler use and stress, anxiety, or worries experienced by patients while using their inhalers, (2) amount of education and help available for patients’ inhaler use, and (3) potential frequency of AR technology use. These core findings are discussed further in the following sections.

#### Confidence, Stress, and Anxiety When Using Inhalers

Despite a lack of knowledge and confidence being mentioned throughout the literature [5,7,21,23,24,26,53,54], it did not appear to be a problem among the health professionals and key community stakeholders interviewed. All health professionals and key community stakeholders who were interviewed (12/12, 100%) expressed confidence in delivering correct inhaler technique and having sufficient knowledge about both the disease and medications. However, during the interviews, all health professionals (8/8, 100%) were questioning themselves as to how adept they really were in communicating this to patients. This suggests that there may be insufficient education readily available and easily accessible for both health professionals and patients with asthma. An incidental yet important finding is the irregular frequency in assessment of inhaler technique with patients, which may not be fully identified as an issue among health professionals until probed. Although health professionals are confident, when quizzed about actual clinical practice, their hesitation suggests that inhaler technique is not addressed sufficiently often during consultations.

Following concerns about lack of knowledge and confidence within the health professional community, all interviewed participants (21/21, 100%) were asked specifically about confidence of patients with asthma while using their inhaler. From the results, health professionals and key community stakeholders perceived patients with asthma as having low levels of confidence during inhaler technique evaluation. However, contrary to these results, all patients with asthma (8/8, 100%) reported feeling confident in using their inhaler. The literature has identified that patients with asthma can be highly confident in performing inhaler technique, despite doing so incorrectly [23,24,55]. A high level of confidence is not a good indicator that inhaler technique will be performed correctly, as highly confident patients make mistakes more or as often as patients who are less confident [23,24]. This suggests that although patients may appear to be confident, health professionals or key community stakeholders still need to regularly review device use. This is likely to lead to more patients who correctly use their asthma inhaler.

Similarly, as patients with asthma perceive themselves as being adept with their inhaler use, they also believe that they do not experience stress, anxiety, or worries while using their medication devices. However, health professionals and key community stakeholders reported that approximately half of the patients with asthma they see will experience some kind of stress, anxiety, or worry related to use of their device at some point in time. This finding may also be linked back to the high confidence level perceived by the patient with asthma, compared with the level observed by the health professionals and key community stakeholders. Patients who do not receive any or sufficient education may be unaware of potential issues with their technique [5,6,18,19,56].

#### Education and Ongoing Assistance for Patient Inhaler Technique

Health professionals agreed that inhaler technique education occurred only on a *when required* basis. In total, 50% (4/8) of the health professionals reported using the assistance of available practice nurses or pharmacists when discussing inhaler technique, to navigate the issues relating to time constraints. In contrast, patients with asthma reported that health professionals rarely provided any education related to inhaler technique. Furthermore, 50% (4/8) of the patients with asthma were unaware of alternative help avenues available for inhaler technique education such as web-based resources and those available via the Asthma Foundation of South Australia. This finding is consistent with the literature [22], suggesting that the limited awareness of available alternative resources stems from the perception of patients with asthma that they do not need additional help. These results support findings in the literature [23,24], suggesting that patients with asthma do not believe that assistance is required and therefore do not seek help. However, opportunities may arise with use of the AR technology resource, not only as a means to increase the amount of available education but also to act as a prompt for patients to intermittently confirm their inhaler technique.

Barriers to regularly reviewing inhaler technique were raised as a concern among all individuals. Similar to previously published studies [5-7,18,19], key barriers around improving inhaler technique included limited appointment time, lack of resources (eg, pamphlets and books), and availability of placebo devices to aid in education. Although the published literature points to time constraints as the greatest limiting factor on correcting inhaler technique, patients with asthma in this study mentioned that other health issues often require attention, leaving minimal or no time to discuss inhaler technique. With this in mind, all participants (21/21, 100%) believed that the AR technology resource could be introduced and implemented during consultation and potentially used as a refresher tool, with the intention of reducing these barriers.

#### Frequency of Smartphone Technology Use

All participants (21/21, 100%) believed that the technology would be beneficial for health professionals and patients with asthma as a means of delivering inhaler education. Following demonstration of the innovative technology, all participants (21/21, 100%) agreed that the handheld resource provides a physical cue that health professionals, patients with asthma, and key community stakeholders are familiar with. Health professionals and key community stakeholders felt that it would be a useful inhaler initiation tool used in conjunction with other support materials. However, patients with asthma believed that the smartphone app would be sufficient without the need for a poster or accompanying physical resource to deliver inhaler technique education as a refresher tool. In this qualitative study, a frequently reported limitation impeding regular use was that some patients with asthma may not use smartphones and, in particular, older people would find it difficult to navigate.

### Future Studies

A randomized controlled trial is needed to evaluate the effectiveness of the AR resource in the clinical care setting. Combined with a brief training program, the physical poster-based resource overlaid with AR technology should be compared with implementation of usual care across multiple sites. Outcomes to evaluate include the following: assessment of correct inhaler technique among patients, number of patients shown inhaler technique by their health professional at each visit, asthma exacerbations, need to use reliever medication, emergency department presentation or hospitalization, and reported use of the electronic resource outside outpatient or hospital visits.

### Limitations

Recruitment was more difficult than initially predicted owing to the paucity of patients with asthma admitted to TQEH who met all the inclusion criteria and were willing to participate. Therefore, more participants with well-controlled asthma were recruited. These participants had fewer exacerbations and better controlled asthma than those originally targeted; however, in the final phase of recruitment, 3 participants fulfilling all the original inclusion criteria were identified and agreed to participate, thus improving generalizability of the findings. For these reasons, the number of recruited patients with asthma is a larger cohort than initially intended. Although unexpected, this broadens the scope of the study to include patients with asthma with well-controlled symptoms and those with frequent hospitalizations.

Another limitation for this study includes the limited representation of diversity in the sample. The sample included people who resided within urban areas of Adelaide, with limited ethnic diversity; had English as a first language; had middle to high socioeconomic status; and were employed. This study would be strengthened with recruitment of a more diverse range of participants from across multiple hospitals and different locations (urban, regional, and rural and across states and territories). Of note, participants were not excluded based on smoking status or time of last lung function assessment. Therefore, a potential exists for some recruited patients with asthma to have undiagnosed early-stage COPD. However, it is unlikely that having undiagnosed COPD will affect the results of the evaluation because patient’s self-identity as a person with asthma will not change.

Purposive sampling used throughout recruitment of patients with asthma poses a risk of participant selection bias that is difficult to measure or control [57]. However, considering that the purpose of this evaluation was to gain insight into specific populations, the targeted sample cohort evaluated in this study is typical of qualitative research. An attempt was made to reduce researcher bias by using 2 independent researchers to code the qualitative data.

As the scope of the paper was limited to themes directly related to the use of new technology as an intervention to improve inhaler technique, it only indirectly addressed the broad strategies for improving asthma inhaler technique, such as pamphlets, face-to-face assessments, and other resources or dynamics used when educating patients.

Methodology used in this study did not incorporate quantitative assessment of inhaler technique used by patients with asthma according to guidelines. Moreover, interviews were conducted using a semistructured guide permitting flexibility in wording and flow of interview. Throughout initial interviews with health professionals, patients with asthma, and key community stakeholders, responses were not as expected in some cases. In these instances, adjustments to the moderator guides occurred, generating more in-depth responses that better addressed the gap in evidence. Counts could not be conducted to quantify responses on any one particular issue. Supplementing this, 10-point Likert scales were used to provide quantitative outputs or key issues that were also used to triangulate data collection.

### Conclusions

Inhaler technique is an important part of asthma management that is frequently overlooked. There is a widely held perception of confidence in inhaler technique among most patients with asthma that may be masking issues of incorrect technique. These perceptions lead to a false sense of security as patients do not ask their health professionals to check inhaler technique, they do not seek resources for confirmation, and health professionals request to see inhaler technique less often. These factors may be exacerbating the high rates of incorrect inhaler technique that are contributing to suboptimal disease management and subsequently escalating hospital use. AR technology was perceived by all participants (21/21, 100%) to be a potentially valuable tool to aid in the education of correct inhaler technique and as a means of prompting health professionals to check inhaler use. The ability to update the digital content easily and cost-effectively without the need to change the printed document is a valuable asset, as is the capacity for videos to negate issues surrounding poor health literacy. Barriers to uptake were identified for the older population who may not know how to use the technology and for people without smartphones. Underevaluation of poor inhaler technique by health professionals was also identified as a potential barrier resulting in reduced uptake of the technology by patients with asthma. As this is a qualitative study, research data cannot be generalized beyond the sample. Therefore, a randomized controlled trial to evaluate efficacy in the clinical setting is required.

## Appendix

Multimedia Appendix 1 Prototype inhaler education poster with augmented reality functionality.

Multimedia Appendix 2 Subthemes and quotes identified under the Triandis model.

## Abbreviations

AR: augmented reality
COPD: chronic obstructive pulmonary disease
GP: general practitioner
TQEH: The Queen Elizabeth Hospital

## References

[1] (2022). Asthma. Australian Bureau of Statistics.

[2] GBD 2019 DiseasesInjuries Collaborators (2020). Global burden of 369 diseases and injuries in 204 countries and territories, 1990-2019: a systematic analysis for the Global Burden of Disease Study 2019. Lancet.

[3] (2021). Global Strategy for Asthma Management and Prevention (2021 update). Global Initiative For Asthma.

[4] Scichilone N (2015). Asthma control: the right inhaler for the right patient. Adv Ther.

[5] Self TH, Arnold LB, Czosnowski LM, Swanson JM, Swanson H (2007). Inadequate skill of healthcare professionals in using asthma inhalation devices. J Asthma.

[6] Basheti I, Hamadi S, Reddel H (2016). Inter-professional education unveiling significant association between asthma knowledge and inhaler technique. Pharm Pract (Granada).

[7] Miles C, Arden-Close E, Thomas M, Bruton A, Yardley L, Hankins M, Kirby SE (2017). Barriers and facilitators of effective self-management in asthma: systematic review and thematic synthesis of patient and healthcare professional views. NPJ Prim Care Respir Med.

[8] Al-Jahdali H, Ahmed A, Al-Harbi A, Khan M, Baharoon S, Bin Salih S, Halwani R, Al-Muhsen S (2013). Improper inhaler technique is associated with poor asthma control and frequent emergency department visits. Allergy Asthma Clin Immunol.

[9] (2016). Global strategy for asthma management and prevention (2016 update). Global Initiative for Asthma.

[10] Zambelli-Simões L, Martins MC, Possari JC, Carvalho GB, Coelho AC, Cipriano SL, Carvalho-Pinto RM, Cukier A, Stelmach R (2015). Validation of scores of use of inhalation devices: valoration of errors. J bras pneumol.

[11] Vanderman AJ, Moss JM, Bailey JC, Melnyk SD, Brown JN (2015). Inhaler misuse in an older adult population. Consult Pharm.

[12] Sadowski CA, Cor K, Cave A, Banh HL (2015). Administration technique and acceptance of inhaler devices in patients with asthma or COPD. Ann Pharmacother.

[13] Price DB, Román-Rodríguez M, McQueen RB, Bosnic-Anticevich S, Carter V, Gruffydd-Jones K, Haughney J, Henrichsen S, Hutton C, Infantino A, Lavorini F, Law LM, Lisspers K, Papi A, Ryan D, Ställberg B, van der Molen T, Chrystyn H (2017). Inhaler errors in the CRITIKAL study: type, frequency, and association with asthma outcomes. J Allergy Clin Immunol Pract.

[14] Basheti IA, Armour CL, Bosnic-Anticevich SZ, Reddel HK (2008). Evaluation of a novel educational strategy, including inhaler-based reminder labels, to improve asthma inhaler technique. Patient Educ Couns.

[15] Crane MA, Jenkins CR, Goeman DP, Douglass JA (2014). Inhaler device technique can be improved in older adults through tailored education: findings from a randomised controlled trial. NPJ Prim Care Respir Med.

[16] Hämmerlein A, Müller U, Schulz M (2011). Pharmacist-led intervention study to improve inhalation technique in asthma and COPD patients. J Eval Clin Pract.

[17] Normansell R, Kew K, Mathioudakis A (2017). Interventions to improve inhaler technique for people with asthma. Cochrane Database Syst Rev.

[18] Torjesen I (2014). Two thirds of deaths from asthma are preventable, confidential inquiry finds. BMJ.

[19] National Review of Asthma Deaths (2015). Why asthma still kills. Royal College of Physicians.

[20] Gagné ME, Boulet L (2018). Implementation of asthma clinical practice guidelines in primary care: a cross-sectional study based on the Knowledge-to-Action Cycle. J Asthma.

[21] Plaza V, Giner J, Rodrigo GJ, Dolovich MB, Sanchis J (2018). Errors in the use of inhalers by health care professionals: a systematic review. J Allergy Clin Immunol Pract.

[22] Cheong LH, Armour CL, Bosnic-Anticevich SZ (2015). Patient asthma networks: understanding who is important and why. Health Expect.

[23] Chorão P, Pereira AM, Fonseca JA (2014). Inhaler devices in asthma and COPD--an assessment of inhaler technique and patient preferences. Respir Med.

[24] Press VG, Arora VM, Shah LM, Lewis SL, Charbeneau J, Naureckas ET, Krishnan JA (2012). Teaching the use of respiratory inhalers to hospitalized patients with asthma or COPD: a randomized trial. J Gen Intern Med.

[25] (2015). Australian Asthma Handbook.

[26] Leung JM, Bhutani M, Leigh R, Pelletier D, Good C, Sin DD (2015). Empowering family physicians to impart proper inhaler teaching to patients with chronic obstructive pulmonary disease and asthma. Can Respir J.

[27] Abu Abed M, Himmel W, Vormfelde S, Koschack J (2014). Video-assisted patient education to modify behavior: a systematic review. Patient Educ Couns.

[28] Wilson EA, Makoul G, Bojarski EA, Bailey SC, Waite KR, Rapp DN, Baker DW, Wolf MS (2012). Comparative analysis of print and multimedia health materials: a review of the literature. Patient Educ Couns.

[29] Wilson M (2009). Readability and patient education materials used for low-income populations. Clin Nurse Spec.

[30] Self TH, Brooks JB, Lieberman P, Ryan MR (1983). The value of demonstration and role of the pharmacist in teaching the correct use of pressurized bronchodilators. Can Med Assoc J.

[31] King T, Kho E, Tiong Y, Julaihi S (2015). Comparison of effectiveness and time-efficiency between multimedia and conventional counselling on metered-dose inhaler technique education. Singapore Med J.

[32] Navarre M, Patel H, Johnson CE, Durance A, McMorris M, Bria W, Erickson SR (2016). Influence of an interactive computer-based inhaler technique tutorial on patient knowledge and inhaler technique. Ann Pharmacother.

[33] Savage I, Goodyer L (2003). Providing information on metered dose inhaler technique: is multimedia as effective as print?. Fam Pract.

[34] van der Palen J, Klein JJ, Kerkhoff AH, van Herwaarden CL, Seydel ER (1997). Evaluation of the long-term effectiveness of three instruction modes for inhaling medicines. Patient Educ Couns.

[35] Mulhall AM, Zafar MA, Record S, Channell H, Panos RJ (2017). A tablet-based multimedia education tool improves provider and subject knowledge of inhaler use techniques. Respir Care.

[36] Zarei AR, Jahanpour F, Alhani F, Razazan N, Ostovar A (2014). The impact of multimedia education on knowledge and self-efficacy among parents of children with asthma: a randomized clinical trial. J Caring Sci.

[37] Iio M, Hamaguchi M, Narita M, Takenaka K, Ohya Y (2017). Tailored education to increase self-efficacy for caregivers of children with asthma: a randomized controlled trial. Comput Inform Nurs.

[38] Bacca-Acosta J, Baldiris S, Fabregat R, Graf S (2014). Augmented reality trends in education: a systematic review of research and applications. Educ Technol Soc.

[39] Kim H, Xie B (2017). Health literacy in the eHealth era: a systematic review of the literature. Patient Educ Couns.

[40] Akçayır M, Akçayır G (2017). Advantages and challenges associated with augmented reality for education: a systematic review of the literature. Educl Res Rev.

[41] Porter ME, Heppelmann JE (2015). How smart, connected products are transforming companies. Harvard Business Review.

[42] Asthma. Lung Foundation Australia.

[43] Layar homepage. Layar.

[44] Cloutier M (2016). Asthma management programs for primary care providers: increasing adherence to asthma guidelines. Curr Opin Allergy Clin Immunol.

[45] Basheti IA, Qunaibi EA, Hamadi SA, Reddel HK (2014). Inhaler technique training and health-care professionals: effective long-term solution for a current problem. Respir Care.

[46] Malterud K, Siersma VD, Guassora AD (2016). Sample size in qualitative interview studies: guided by information power. Qual Health Res.

[47] Palinkas LA, Horwitz SM, Green CA, Wisdom JP, Duan N, Hoagwood K (2015). Purposeful sampling for qualitative data collection and analysis in mixed method implementation research. Adm Policy Ment Health.

[48] Andersen KH (2007). Nothing is as practical as a good theory. Fish Ecology, Evolution, and Exploitation.

[49] Patton MQ (1999). Enhancing the quality and credibility of qualitative analysis. Health Serv Res.

[50] Darnton A (2008). Reference report: an overview of behaviour change models and their uses. GSR Behaviour Change Knowledge Review.

[51] Bamberg S, Schmidt P (2016). Incentives, morality, or habit? Predicting students’ car use for university routes with the models of Ajzen, Schwartz, and Triandis. Environ Behav.

[52] Robinson J (2010). Triandis’ Theory of Interpersonal Behaviour in understanding software piracy behaviour in the South African context. CORE.

[53] Fink J, Rubin B (2005). Problems with inhaler use: a call for improved clinician and patient education. Respir Care.

[54] Lindh A, Theander K, Arne M, Lisspers K, Lundh L, Sandelowsky H, Ställberg B, Westerdahl E, Zakrisson A (2019). Errors in inhaler use related to devices and to inhalation technique among patients with chronic obstructive pulmonary disease in primary health care. Nurs Open.

[55] Usmani OS, Lavorini F, Marshall J, Dunlop WC, Heron L, Farrington E, Dekhuijzen R (2018). Critical inhaler errors in asthma and COPD: a systematic review of impact on health outcomes. Respir Res.

[56] Soones TN, Lin JL, Wolf MS, O'Conor R, Martynenko M, Wisnivesky JP, Federman AD (2017). Pathways linking health literacy, health beliefs, and cognition to medication adherence in older adults with asthma. J Allergy Clin Immunol.

[57] Acharya AS, Prakash A, Saxena P, Nigam A (2013). Sampling: why and how of it?. Indian J Med Spec.

