# Supplementary material for: An Augmented Reality Technology to Provide Demonstrative Inhaler Technique Education for Patients With Asthma: Interview Study Among Patients, Health Professionals, and Key Community Stakeholders
Source: JMIR Form Res. 2023 Mar 2;7:e34958. doi: 10.2196/34958 (PMC10020912; doi:10.2196/34958)
Supplement: Multimedia Appendix 1 [file formative_v7i1e34958_app1.docx]

**Multimedia Appendix 1: Prototype inhaler education poster with augmented reality functionality**

**
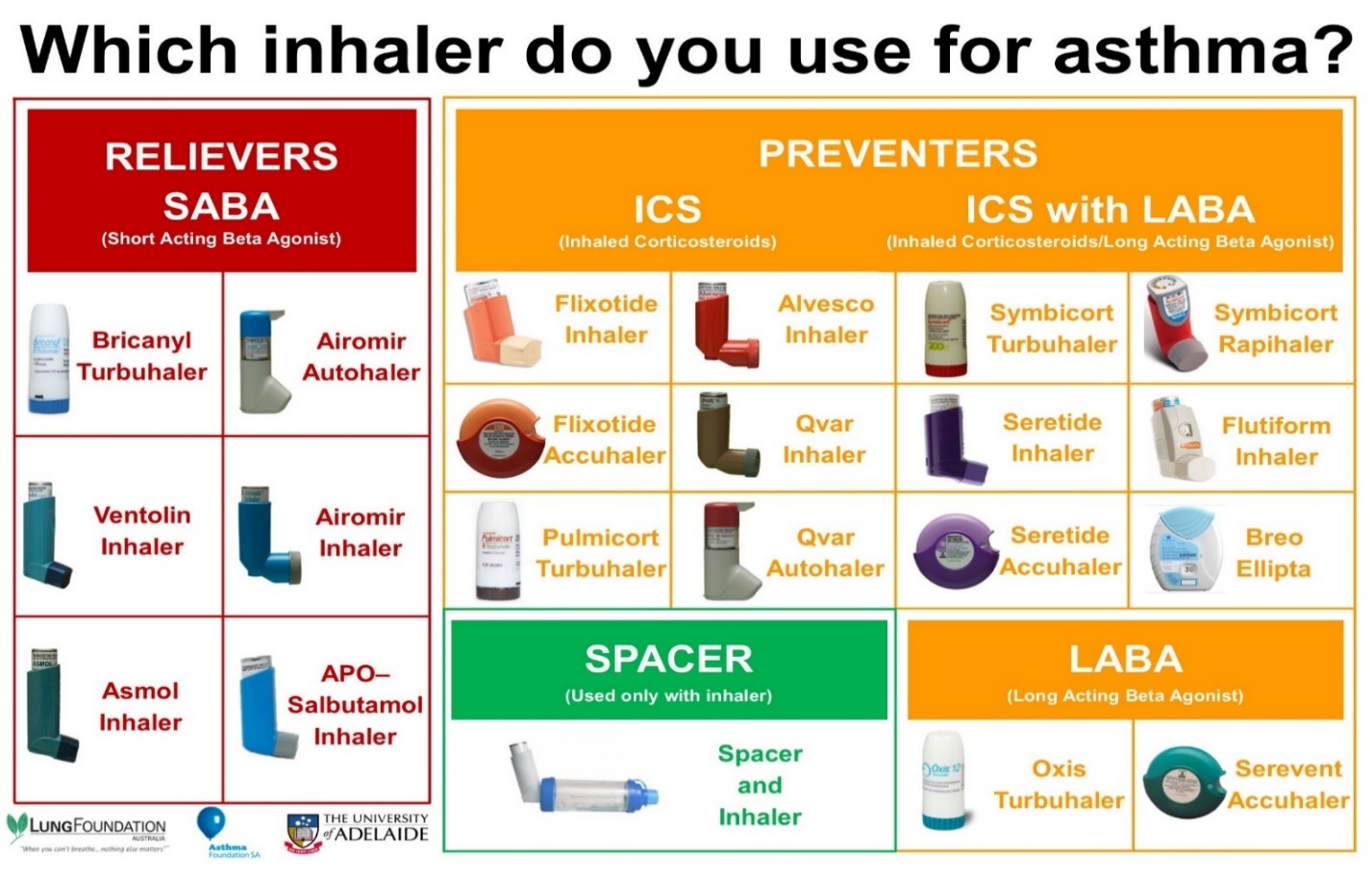
**
