# Supplementary material for: An Augmented Reality Technology to Provide Demonstrative Inhaler Technique Education for Patients With Asthma: Interview Study Among Patients, Health Professionals, and Key Community Stakeholders
Source: JMIR Form Res. 2023 Mar 2;7:e34958. doi: 10.2196/34958 (PMC10020912; doi:10.2196/34958)
Supplement: Multimedia Appendix 2 [file formative_v7i1e34958_app2.docx]

**Multimedia Appendix 2:** Subthemes and quotes identified under the Triandis model.

| Perspectives and Triandis levels | | Subcategories | Quotes |
| --- | --- | --- | --- |
| **Positive** | | | |
|  | **First level** | | |
|  |  | Demonstration of device | - “Well he dug out the spacer and out the thing on the end and said well what you do is you know, press, breathe in and he watched me do it and he said yeah that’s fine.” [AP^a^ 6] - “...If I do have a demo inhaler in my clinic I’ll show them how to use it or sometimes I feel it’s easier to just play a video on YouTube if time permits as well during the clinic session.” [HP^b^ 2] |
|  |  | Checking inhaler technique | - “Well the best way and the one that I prefer the most is hands on...we actually sit in with the person with either a placebo or their own puffers and actually doing it, re-enacting, so an aesthetic approach.” [KCS^c^ 4] - “Not every time but some of the times when I’ve been back for a prescription renewal and they’ve said make sure you do this and wash your mouth out afterwards and more often than not they remind me.” [AP 3] |
|  |  | Teaching correct inhaler technique | - “We provide inhaler technique education beyond asthma...we do it to all your respiratory chronic diseases and that would be part of most of our consults...that would be...making sure that people are using their devices correct.” [HP 5] |
|  | **Second level** | | |
|  |  | Confidence in delivery of education | - “I think I’ve got the knowledge, I feel confident with it...because I’ve been asthmatic...and I deal a lot...with some of these asthma kids from up at the hospital...I feel pretty good with that because I’ve seen enough of it.” [HP 4] |
|  |  | Responsibility for delivery of inhaler technique education | - “Probably doctors but the lung specialist at the moment has probably been the best one.” [AP 5] - “At the moment the practice nurse would be the one that would do it.” [AP 6] - “It would be good if firstly the doctor could explain that to them and pharmacists as well. If you have more people teaching in a collaborative manner that will help reinforce the techniques because I think people learn by repetition.” [HP 8] |
|  |  | Applicability of technology | - “Most people are able to press a few buttons...most people would use it. If you’ve got a young mum out there...she’s concerned about her child that has asthma and this can show her exactly...how to use the puffer which makes it brilliant.” [KCS 1] |
|  | **Third level** | | |
|  |  | Development of habit | - “For some people like they may develop habit but it may be that it’s working for them, they are still getting that benefit from the medications...their symptoms are controlled, they’re taking it, taking their medications at the regular intervals that is required.” [HP 7] |
|  |  | Level of care that patients have | - “I think most patients that I’ve seen...they do care...especially mums with young children that are just starting it.” [HP 7] - “If I get a new puffer I can check it up or and see if the doctors not actually lying to me or something.” [AP 5] |
|  |  | Good habits | - “I believe that if the first time they’ve already been taught the right way then it becomes a habit of them.” [HP 6] - “Like a golf swing, it’s like if you...have a good teacher initially uhm a doctor or a pharmacist that shows you how to do it properly and you repeat that...good habits will develop and your asthma as a result of that will be better controlled.” [HP 8] |
| **Negative** | | | |
|  | **First level** | | |
|  |  | Formal education about disease, medications, and devices | - “I think some pharmacists won’t check that it’s new or their dispense techs don’t tell them that it is new and then it just gets missed...” [HP 7] - “I even still don’t really fully understand the scientific side of asthma and the disease. I know the basics but it’s never, I’ve never attended anything that’s really explained to me what it mean...how it impacts my body...I was basically just given a puffer and told this will fix it not knowing whether I was going to have it for life.” [AP 2] |
|  |  | Beliefs about outcomes | - “Time is a major barrier. So obviously clinics are full packed and jammed so don’t have much time.” [HP 1] - “Honestly not everyone I’d talk to about inhaler technique...it’s super important, like I know I’ve gotta get better at it...but I think we all take it for granted that they know what to do.” [HP 4] |
|  |  | Assumptions regarding confidence with using an inhaler correctly | - “I think patients just assume they’re using their inhalers correctly...because it’s a bit, oh how hard could this be...but there might be subtle things that are not right.” [HP 3] - “Cause you get lazy at these things...people take shortcuts.” [HP 4] - “People tend to think they know everything and sometimes they’re not that willing to listen so often...you’ll say to somebody, can you show me how you use your medication, and they’ll tell you, oh I know how to use it, I use it correctly and...you can’t tell them anything that they don’t already know.” [KCS 3] |
|  | **Second level** | | |
|  |  | Responsible for inhaler education delivery | - “It’s a shared responsibility...I just wouldn’t probably delegate it to the prescriber necessarily...but because we don’t make it someone’s absolute responsibility therefore patients fall between the cracks...” [HP 3] |
|  |  | Abundance of varying instructions | - “People are just giving different instructions...this is how I’ve been told, this is how the doctor so that’s how I give and then there’s other staff coming into follow up and it’s a different technique.” [HP 6] - “There’s different opinions between different doctors and then some doctors stick with only one type and then it, you don’t even know, I mean how the hell is someone like me supposed to know.” [AP 2] |
|  |  | Importance of inhaler technique | - “When you’ve got busy lives and everything, the importance of medication sort of forgets put to the, you know, background or whatever and you’re not really thinking about it until now.” [HP 5] - “It’s a big issue because you’ve got to get the drugs into your lungs and if you don’t get the drugs into your lungs you don’t have good control.” [AP 6] |
|  | **Third level** | | |
|  |  | Beliefs that inhalers are not working | - “I feel that people neglect the use of preventer’s because they don’t give immediate benefit from shortness of breath...education on the underlying inflammation...and the benefits of using a corticosteroid inhaler...knowing that...preventers...prevent the onset of asthma attacks...” [HP 8] |
|  |  | Age at diagnosis and effect on inhaler technique education | - “Maybe people like myself who get diagnosed really young and perhaps they’re...not personally educated. Their parents have been educated and then there’s no sort of update on them being educated.” [AP 4] |
|  |  | Problems affecting proper inhaler technique | - “Their health literacy’s like and things like that come into play as well...if they’re not understanding so much about how important all these things are...if they can’t follow instructions...” [HP 5] - “A lot of people just think I manage my asthma by using my Ventolin...I don’t like using my preventer and I certainly wouldn’t use a spacer...so there are a lot of issues when for people when we’re trying to extend outreach and impact actually get them to take their condition quite seriously.” [KCS 2] |

^a^AP: patient with asthma.

^b^HP: health professional.

^c^KCS: key community stakeholder.
